# Analysis and Design of a 3rd Order Velocity-Controlled Closed-Loop for MEMS Vibratory Gyroscopes

**DOI:** 10.3390/s130912564

**Published:** 2013-09-18

**Authors:** Huan-ming Wu, Hai-gang Yang, Tao Yin, Ji-wei Jiao

**Affiliations:** 1 Institute of Electronics, Chinese Academy of Sciences, Beijing 100190, China; E-Mails: guantou728@gmail.com (H.W.); yint@mail.ie.ac.cn (T.Y.); 2 Ningbo High-tech Institute, Ningbo University, Ningbo 315211, China; 3 Shanghai Institute of Microsystem and Information Technology, Chinese Academy of Sciences, Shanghai 200050, China; E-Mail: jiaojw@mail.sim.ac.cn

**Keywords:** MEMS vibratory gyroscopes, velocity-controlled closed-loop, 3rd order, linear model, optimization methodology

## Abstract

The time-average method currently available is limited to analyzing the specific performance of the automatic gain control-proportional and integral (AGC-PI) based velocity-controlled closed-loop in a micro-electro-mechanical systems (MEMS) vibratory gyroscope, since it is hard to solve nonlinear functions in the time domain when the control loop reaches to 3rd order. In this paper, we propose a linearization design approach to overcome this limitation by establishing a 3rd order linear model of the control loop and transferring the analysis to the frequency domain. Order reduction is applied on the built linear model's transfer function by constructing a zero-pole doublet, and therefore mathematical expression of each control loop's performance specification is obtained. Then an optimization methodology is summarized, which reveals that a robust, stable and swift control loop can be achieved by carefully selecting the system parameters following a priority order. Closed-loop drive circuits are designed and implemented using 0.35 μm complementary metal oxide semiconductor (CMOS) process, and experiments carried out on a gyroscope prototype verify the optimization methodology that an optimized stability of the control loop can be achieved by constructing the zero-pole doublet, and disturbance rejection capability (D.R.C) of the control loop can be improved by increasing the integral term.

## 1. Introduction

Due to their wide applications in inertial navigation, automotive stability control and robots, MEMS gyroscopes have drawn tremendous attention of researchers in both academia and industry [1–4]. Most MEMS gyroscopes are based on the Coriolis force effect. The Coriolis force is proportional to the angular rate only on the premise of a constant vibration velocity in the drive axis. As a result, automatic gain control (AGC) based velocity-controlled closed-loops are commonly built to keep the primary resonator vibrating at its resonant frequency with constant amplitude [5–12]. The velocity-controlled closed-loop always introduces stability problems, which are the primary concern in its design process.

The time-average method is widely applied in the stability analysis of control loops [7,9,13–15]. In the time-average method, the controlled amplitude is considered as a constant in one vibratory period to simplify the nonlinear equations of the control loop, since the variation rates of the controlled amplitude are usually much slower than the vibratory rate. In [9,13], the nonlinear equations are simplified by using the time-average method and the effects of AGC parameters on the response of the linearized dynamics are revealed. Similar work is also done in [14,15], which use a Lyapunov function to obtain a good transient response. However, in all these works, no quantitative stability criterion is given. In [7], a nonlinear mathematical model in time domain of a AGC-PI closed-loop is linearized by firstly using a time-average method. Then the mathematical expression of the stability criterion is derived by applying the Routh-Hurwitz criterion to the characteristic equations. Specific transient response performance is discussed when the control loop is a 2nd order system with only the proportional controller. In reality, in order to improve the loop control accuracy, an integral function is imported into the controller of the closed-loop, and the AGC-PI closed-loop structure is widely used in the driving circuit of MEMS vibratory gyroscopes which is a 3rd order system. Nevertheless, the specific transient response performance of a 3rd order system can hardly be attained using the time-average method due to the difficulties in solving the complicated nonlinear functions [7]. In summary, the time-average method has two main drawbacks: firstly, the entire stability analysis process is tedious and exhausting as it solves nonlinear functions “in the time domain”; secondly, it can not be used to analysis the specific performance of the 3rd order controlled loop.

To overcome these two drawbacks of the time-average method mentioned above, a linearization design approach is presented in this paper. A fully linear system model of the 3rd order closed-loop is established and thereafter the stability and performance are analyzed in the frequency domain. Compared to the conventional time-average method, the proposed linearization design approach has the following advantages: first, the stability criterion of the closed-loop can be simply obtained by applying a zero-pole method on the linear model; second, the mathematical expression of each performance specification is obtained by applying order reduction on the 3rd order linear model, so that the dilemma in [7] mentioned above is solved; third, an optimization methodology is summarized, which reveals that a robust, stable and swift control loop can be achieved by carefully selecting the system parameters following a priority order. The proposed optimization methodology is verified by both numerical simulations and experiments.

This paper is organized as follows: Section 2 presents the overall linearization design approach, including building the linear model, revealing the stability criterion, analyzing the control loop's performance through mathematical expressions and numerical simulation results, and summarizing an optimization methodology. In Section 3, the implemented AGC-PI based closed-loop drive circuits are introduced briefly and detailed experimental results on a gyroscope prototype are presented to verify the proposed optimization methodology. Finally, a conclusion is given in Section 4.

## 2. Linearization Design Approach

**Figure 1.**
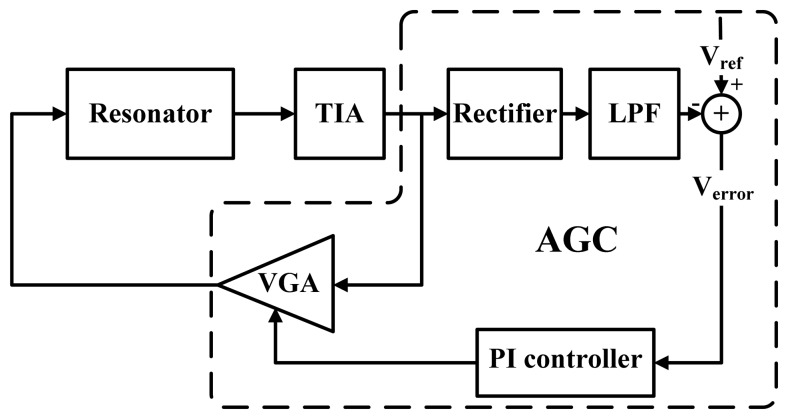
AGC-PI based velocity-controlled closed-loop for the drive mode of MEMS vibratory gyroscope.

The topology of the AGC-PI based velocity-controlled closed-loop for the drive mode of MEMS gyroscope is shown in Figure 1. The displacement of sense comb fingers induces an alternating current, which is converted to voltage by the trans-impedance amplifier (TIA). The voltage is fed into the variable gain amplifier (VGA) to generate the excitation signal. The TIA, VGA and primary resonator of the gyroscope form an electromechanical oscillator. The vibratory velocity of this electromechanical oscillator is controlled to a target value *V_ref_* by an AGC, which consists of rectifier, low pass filter (LPF), proportional and integral (PI) controller and VGA. In the AGC, the rectifier and LPF extract the amplitude information of the vibratory velocity and then compare it with the target value *V_ref_* to obtain an error signal *V_error_*. The error signal is amplified and integrated by the PI controller and then transmitted to adjust the gain of the VGA. The control loop achieves a steady state until the error signal is zero.

The kinetic equation of the drive mode in gyroscope is described as:

(1) $$\ddot{x}+\frac{\omega_{x}}{Q}\dot{x}+\omega_{x}^{2}x=\frac{F_{\text{ext}}}{m_{x}}$$

where *x* is the displacement of the resonator, *ω_x_* is the natural frequency, *Q* is the quality factor, *m_x_* is the mass and *F_ext_* is the driving force.

### 2.1. Linear System Model

**Figure 2.**
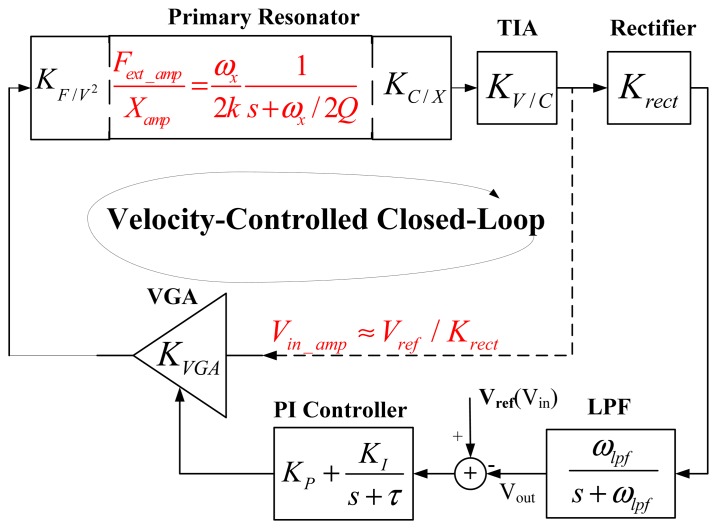
3rd order linear system model of the velocity-controlled closed-loop.

The velocity-controlled closed-loop is a nonlinear system, which processes the amplitude information of the sinusoidal resonance signal. It is hard to analyze the behavior of a nonlinear system, especially when the system usually reaches a 3rd order. Fortunately, a 3rd order linear model of the closed-loop can be built by linearizing two nonlinear modules in the loop, as shown in Figure 2. In the figure, *K_C/X_* and *K_F/V_^2^* model the gain from displacement to capacitance and the gain from the squared excitation voltage to the force, respectively. *K_V/C_* models the gain of capacitance-to-voltage in TIA and *K_VGA_* represents the gain coefficient of VGA. In the amplitude information control path, a rectifier detects the envelope of the resonance signal with gain *K_rect_*, and then the LPF and PI controller process the amplitude signal with their correspondence transfer functions.

The nonlinearity of the closed-loop generates arises from two modules: the primary resonator and the VGA. As the phase perturbation term is negligible compared with *ω_x_*/*(2Q)* in most MEMS vibratory gyroscopes, Equation (1) can be reduced to a first-order system [5], expressed as:

(2) $$\frac{X_{\text{amp}}}{F_{\text{ext}\_\text{amp}}}=\frac{\omega_{x}}{2k}\frac{1}{s+\omega_{x}/2Q}$$

where *F_ext_amp_* and *X_amp_* is the amplitude of *F_ext_* and *x* respectively, *k* is the spring constant. The nonlinearity of the VGA comes from the multiplication of its input and the gain control signal, both of which reflect the amplitude information of the vibration velocity. When the control loop gain is high and the perturbation term is small compared to the velocity amplitude, the error signal is close to zero and the magnitude of the VGA input *V_in_amp_* can be approximated as *V_ref_/K_rect_* [8]. As a result, with respect to the amplitude information control, the electromechanical resonating loop is broken at the VGA's input and only a linear velocity-controlled loop exists.

The control loop is a negative feedback system. As shown in Figure 2, defining the small signal ac component of the reference voltage *V_ref_* as input *V_in_* and the amplitude of the vibration velocity as output *V_out_*, the closed-loop transfer function can be illustrated as:

(3) $$\frac{V_{\text{out}}}{V_{in}}=\frac{H_{\text{loop}}(s)}{1+H_{\text{loop}}(s)}$$

where the feedback factor is 1, and the open-loop transfer function of the linearized control loop can be expressed as:

(4) $$H_{\text{loop}}(s)=K_{F/V^{2}}K_{C/X}K_{V/C}K_{\text{VGA}}V_{\text{ref}}V_{dc}\frac{\omega_{x}}{2k}\cdot \frac{1}{s+\omega_{x}/(2Q)}\cdot \frac{\omega_{\text{lpf}}}{s+\omega_{\text{lpf}}}\cdot \left(K_{P}+\frac{K_{I}}{s+\tau }\right)$$

where *V_dc_* is the DC voltage across the drive comb fingers, *ω_lpf_* is the pole of LPF, *K_P_* is the proportional term and *K_I_* is the integral term in the controller, τ is the loss of integrator.

The built linear system model shown in Figure 2 can be extended to arbitrary higher-order control loops by obeying the following rule: calculating all the significant zeros and poles into the linear model, which usually are located within ten times the control loop's bandwidth.

### 2.2. Stability Criterion

**Figure 3.**
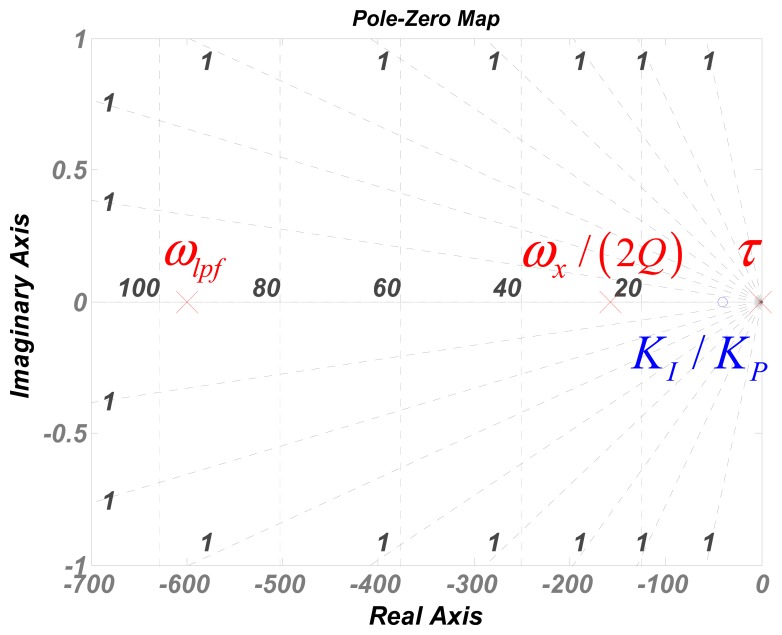
Zero-pole map of the 3rd order velocity-controlled closed-loop.

Figure 3 shows the three poles and the single negative zero in Equation (4). Based on the zero-pole method, as the phase margin compensation characteristic of negative zero, the zero should be located within the largest pole among the three to promise a stable loop. Therefore, the stability criterion of the control loop can be simply written as:

(5) $$\frac{K_{I}}{K_{P}}<\max(\omega_{\text{lpf}},\frac{\omega_{x}}{2Q},\tau )$$

### 2.3. Optimization Methodology

Specifications including loop gain, bandwidth and phase margin are commonly used to evaluate the performances of a negative feedback system in the frequency domain. Mapping these specifications to the provided control loop, their influence on the control loop's performances is illustrated as follows. The loop gain determines the disturbance rejection capability of the control loop against the parameter variance in both mechanical structures and circuits. The bandwidth determines the recovery speed of the control loop and the phase margin reflects the stability of the control loop. These specifications correspond with transient response specifications in the time domain in [7], referred to as setting time, rising time and overshooting. The mathematical expressions of these three performance specifications are derived as follows: firstly, the loop gain of the control loop can be obtained by substituting *s* = 0 in Equation (4), expressed as:

(6) $$H_{\text{loop}}(0)=K_{\text{total}}\frac{2Q}{\omega_{x}}\cdot \left(K_{P}+\frac{K_{I}}{\tau }\right)$$

where:

(7) $$K_{\text{total}}=K_{F/V^{2}}K_{C/X}K_{V/C}K_{\text{VGA}}V_{\text{ref}}V_{dc}\cdot \frac{\omega_{x}}{2k}$$

The expressions of the bandwidth and phase margin can be calculated by solving a third-order equation generated from Equation (4). However, the solution process is very complex. Let the negative zero *K_I_*/*K_P_* equal to the pole *ω_x_/(2Q)*. As a result, a zero-pole doublet is constructed and the 3rd order system is reduced to a second-order system. The validity of the order reduction will be revealed in following numerical simulations. The order-reduced transfer function from Equation (4) can be rewritten as:

(8) $$H_{\text{loop}}(s)=K_{\text{total}}\cdot \frac{\omega_{\text{lpf}}}{s+\omega_{\text{lpf}}}\cdot \frac{K_{P}}{s}$$

in which the integrator is considered as ideal and its loss is ignored. Then the expression of bandwidth a phase margin can be calculated as:

(9) $$BW=\frac{\omega_{\text{lpf}}\left(\sqrt{1+4K_{\text{total}}K_{P}/\omega_{\text{lpf}}}-1\right)}{2}$$

and:

(10) $$PM=\frac{\pi }{2}-\arctan\left(\frac{\sqrt{1+4K_{\text{total}}K_{P}/\omega_{\text{lpf}}}-1}{2}\right)$$

According to Equation (6), Equations (9) and (10), each performance specification variation trend *versus* the primary system parameters are summarized in Table 1. As shown in the table, the performance specifications of the control loop contradict each other. Increasing the loop gain improves the disturbance rejection capability and the control accuracy of the system, while sacrificing the phase margin as well as the stability. Extending the bandwidth increases the response speed of the control system, but may reduce the phase margin and cause an unstable oscillation. As a result, some trade-offs should be made to achieve a precise, stable and swift control loop.

The order of the consideration on the performance specification optimization is usually loop gain > phase margin > bandwidth. The reason can be illustrated as follows: because the mechanical parameter variations are dominant in the control loop and most parameters of the primary resonator, like quality factor *Q*, fluctuate slowly but with a wide range [16]. To suppress these mechanical parameter variations and maintain a constant vibration, the loop gain of the control loop should be considered first. When the gyroscope is under an external disturbance, a low phase margin of the control loop would introduce a large overshoot and cause a large fluctuation at the sense part of the gyroscope. As a result, the phase margin should be considered secondly and the bandwidth is the last.

Numerical simulation results of both the 3rd order control loop linear model and corresponding nonlinear model are provided in the following paragraphs for three reasons: first, it is intended to reveal the validity of the order-reduction; second, it is used to verify the derived results in Table 1; third, it is used to supply the effects of parameters *K_I_* and *Q* on bandwidth and phase margin, which are missing in Table 1 due to the order-reduction.

The parameters of the control loop used in the simulations are listed in Table 2, where *V_p_* is the biased voltage on the movable mass of the resonator and *V_mid_* is the biased voltage on the drive comb fingers. The difference of *V_p_* and *V_mid_* determines the *V_dc_* in Equation (4). To compare the simulation results with experiment results, the resonance frequency *ω_x_* and quality *Q* are especially chosen the same as ones in experiments.

**Figure 4.**
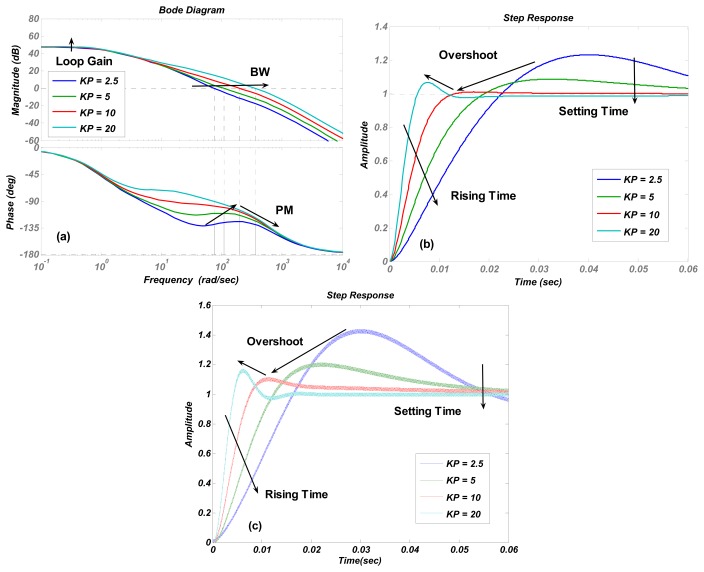
(**a**) Bode diagrams of the control loop (**b**) step responses of the linear model (**c**) and step responses of nonlinear model varying with the proportional term *K_P_*.

**Figure 5.**
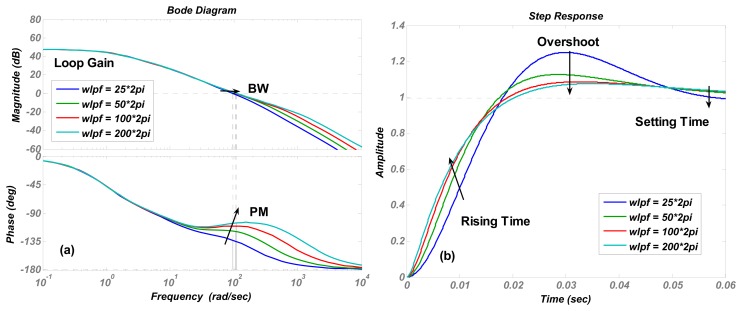

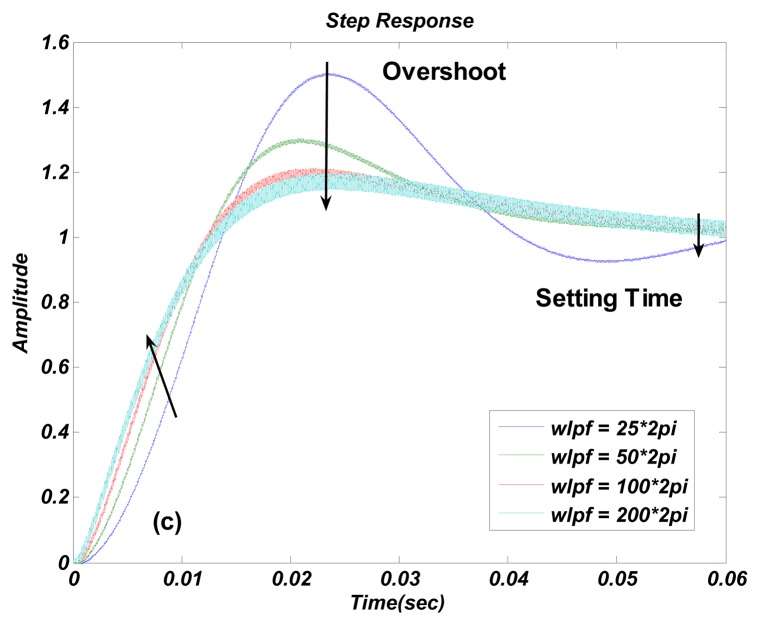
(**a**) Bode diagrams of the control loop (**b**) step responses of the linear model (**c**) and step responses of nonlinear model varying with the cut-off frequency *ω_lpf_*.

**Figure 6.**
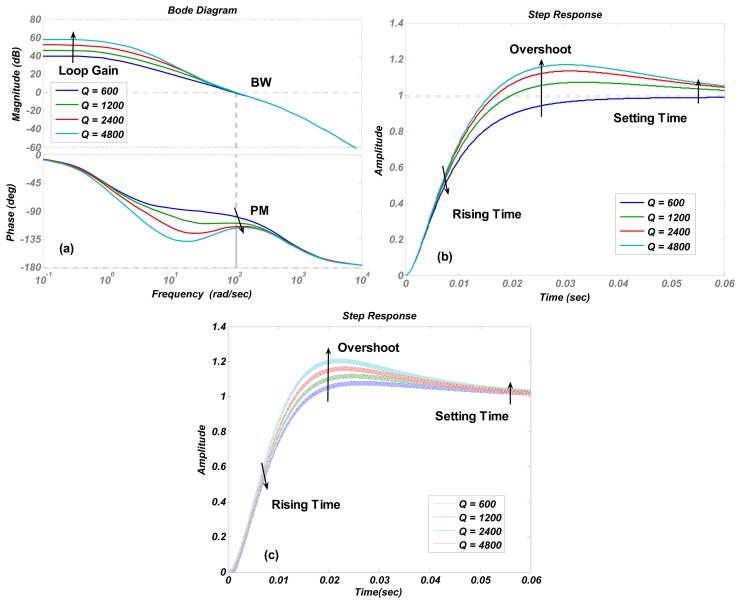
(**a**) Bode diagrams of the control loop (**b**) step responses of the linear model (**c**) and step responses of nonlinear model varying with the integral term *K_I_*.

**Figure 7.**
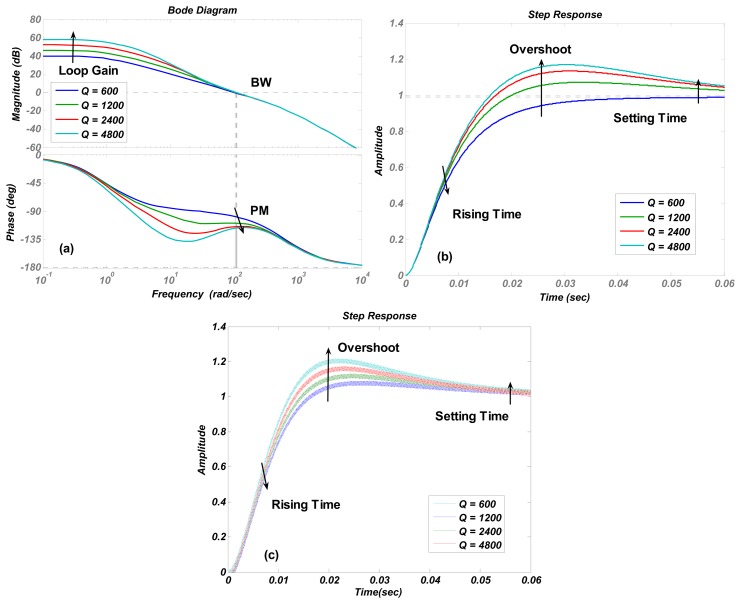
(**a**) Bode diagrams of the control loop (**b**) step responses of the linear model (**c**) and step responses of nonlinear model varying with the quality factor *Q*.

**Figure 8.**
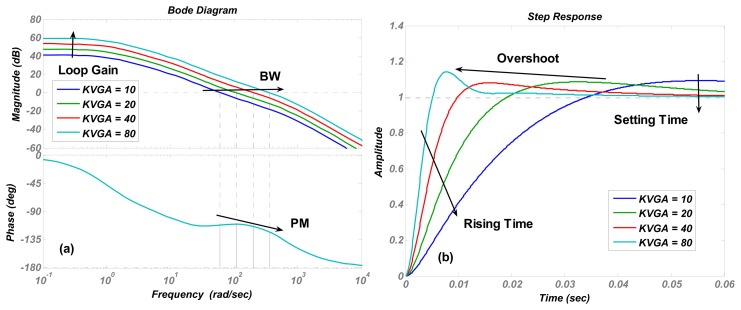

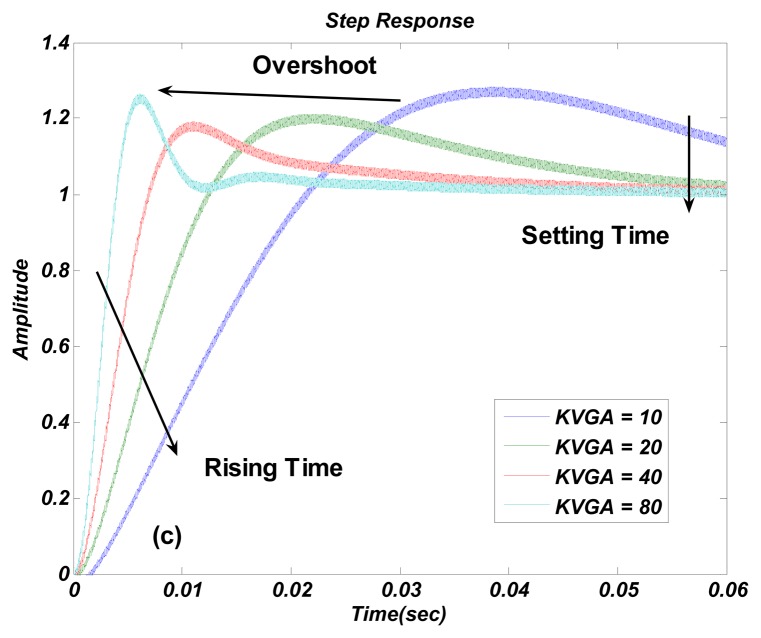
(**a**) Bode diagrams of the control loop (**b**) step responses of the linear model (**c**) and step responses of nonlinear model varying with the VGA gain coefficient *K_VGA_*.

The numerical simulations are carried out in Matlab. The simulation results of the transfer function in Equation (4) are shown in Figures 4, 5, 6, 7 and 8a with varying *K_P_*, *ω_lpf_*, *K_I_*, and *Q*. The corresponding step responses at the LPF output terminal of the proposed closed-loop linear model are also given in Figures 4, 5, 6, 7, and 8b. To verify the validity of the proposed linear model, a nonlinear model is built by Simulink and step responses at the LPF output terminal of corresponding nonlinear model are shown in Figures 4, 5, 6, 7, and 8c. As a 1st-order low pass filter is used in the nonlinear model, the curves in Figures 4, 5, 6, 7, and 8c contain residual resonance-frequency related components and seem noisy.

The validity of the order-reduction is proved in Figure 4. In Figure 4a, the variation trend of the phase margin first increases and then decreases when *K_P_* is doubled. It is noticed that the best phase margin is achieved when the zero-pole doublet is created at *K_P_* = 10, referring to the resonator parameters *ω_x_* and *Q* in Table 2. This result indicates that the optimized stability of the control loop is achieved when the zero-pole doublet is created, and therefore demonstrates that the validity of the order-reduction. The smallest overshoot in Figure 4b at *K_P_* = 10 also verifies the conclusion.

Figure 6a shows that increasing the proportional term *K_I_* is an effective way to enlarge the loop gain. However, it causes an obvious decrease in the phase margin and only a little increment in bandwidth. The trends of curves in Figure 7a are identical to those in Figure 6a, but the phase margin and bandwidth variation quantity is less than those in Figure 6a, which indicates that the stability and recovery speed of the control loop is more robust under the variation of quality factor *Q* than that of *K_I_*. It is believed that the phenomenon is decided by zero and poles locations. This conclusion is verified by simulations with different parameter settings, but not shown as the long paragraph. The curve trends in other figures all agreed with the results in Table 2.

The step responses of the nonlinear model in Figures 4, 5, 6, 7, and 8c are similar to those of the linear model in Figures 4, 5, 6, 7, and 8b. However, the simulation responses of the nonlinear model have shorter rise time and larger overshoot than those of the linear model. The difference is probably caused by fixing VGA input as its final value *V_ref_*/*K_rect_*, which dismisses the action of drive loop in the actual nonlinear model.

Combining the theoretical and simulation results with the specification optimization order presented above, an optimization methodology of system parameters selection can be summarized as follows: to achieve a robust, stable and swift control loop, it is better to increase *K_I_* or *K_VGA_* to achieve a large loop gain and good disturbance rejection capability first, and then pursue an optimized stability by creating a zero-pole doublet through adjusting *K_P_*.

## 3. Circuit Design and Experiments

**Figure 9.**
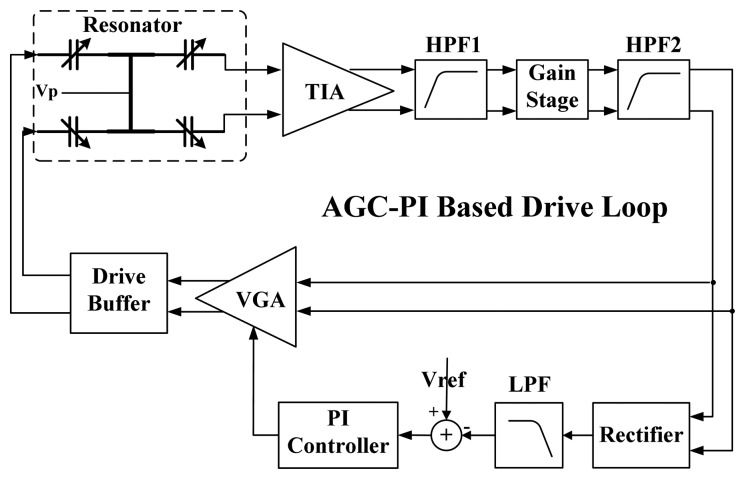
Block diagram of the implemented AGC-PI based closed-loop drive circuits.

To verify the proposed optimization method on a practical gyroscope prototype, AGC-PI based closed-loop drive circuits with flexible system parameters adjustment are designed. The block diagram is presented in Figure 9. In the drive circuits, an adjustable gain stage is designed to match the mechanical gain variation of different resonators. The PI controller adopts a Gm-C structure, which provides convenient and sufficiently tunable properties of *K_P_* and *K_I_* [17]. Two high pass filters (HPFs) are designed for DC offset cancellation.

**Figure 10.**
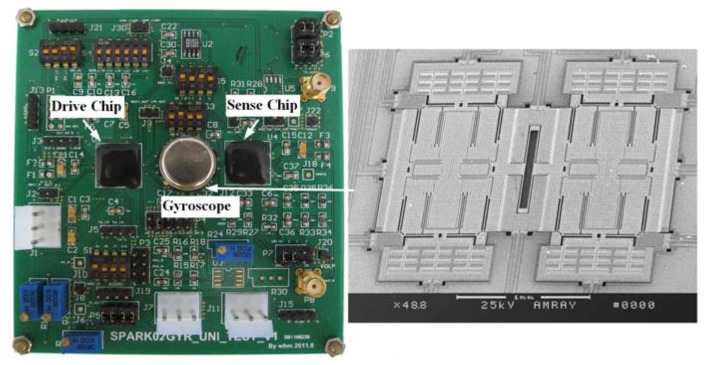
Test board of the gyroscope prototype.

The drive circuits are implemented in a 0.35 μm CMOS technology. The supply voltage is 5 V. A symmetrical doubly decoupled z-axis gyroscope is tested, which adopts an electrostatical drive and capacitive sense. The gyroscope is vacuum-packaged and mounted on a printed circuit board (PCB) with the implemented drive circuits chip and other necessary external resistances and capacitances, as shown in Figure 10. The resonant frequency and the quality factor of the resonator are measured as 8.063 kHz and 1,368, respectively.

To verify the proposed optimization methodology accurately, the velocity-controlled closed-loop should be tested working as a linear system during the experiments. Carefully investigating the linear model, the input of the VGA module is approximated as the final steady value *V_ref_*/*(2*/*π)*. However, in the start-up period, the input of the VGA is nearly zero and the gain of VGA is fixed at the maximum value due to the overlarge error signal fed into the PI controller. As a result, the velocity-controlled closed-loop is broken at the VGA module and the analysis on the linear model above fails. Therefore, snatching start-up waveforms as described in [7] cannot be used in our work.

**Figure 11.**
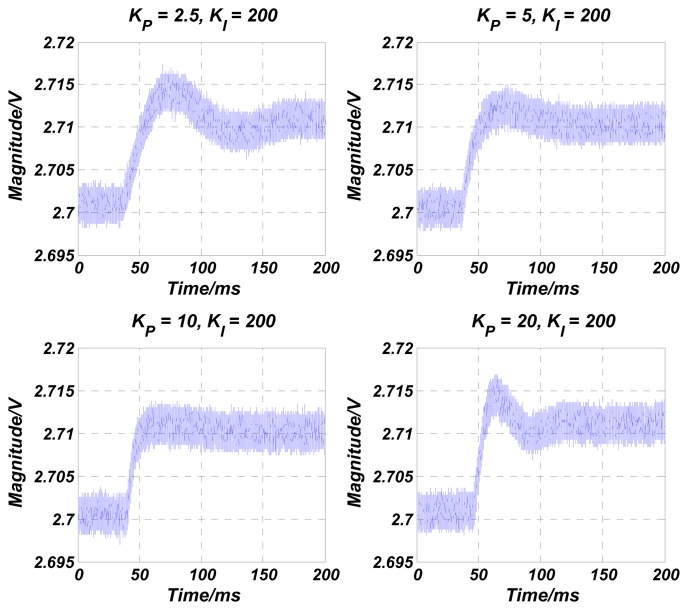
Measured step-up waveforms of the LPF's output with different proportional terms *K_P_*.

**Figure 12.**
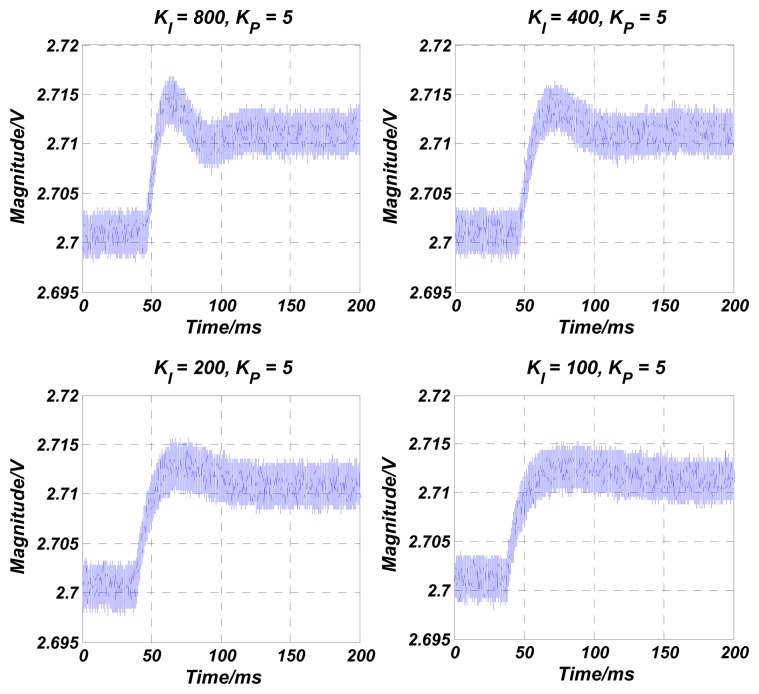
Measured step-up waveforms of the LPF's output with different the integral term *K_I_*.

In our experiments, a 10 mV step-up signal is applied on the target voltage *V_ref_* when the control loop is in a steady state. As the step is very small, the VGA input signal can be considered as a constant and the velocity-controlled closed-loop is believed to work as a linear system. The LPF's output waveform, which reflects the vibration velocity of the resonator, is then recorded with a varied proportional term *K_P_* and integral term *K_I_*. *K_P_* and *K_I_* in the PI controller are decreased gradually by changing the off-chip resistances and capacitances [14]. Figure 11 shows the measured step-up waveforms of the LPF's output varied with *K_P_*. As *K_P_* is increased from 2.5 to 10, the overshoot is smoothed gradually, and the rising time and the setting time are improved. When *K_P_* is set as 20, the overshoot is increased as expected. The test results prove that an optimized stability of the control loop is achieved at situation of *K_P_* = 10 and *K_I_* = 200, when a zero-pole doublet is almost formed, referring to the tested resonators' parameters above. This is exactly the same as the simulation result. The tested rising times are given in Table 3 and compared with the simulation results of both linear and nonlinear models in Figure 4b,c. It is seen that the simulated nonlinear model's rising times are identical with the tested results. As expected, due to the approximation in VGA during the linearization, the rising times of linear model show some “delay” compared with the test results. However, their variation trends are the same, which also verifies the conclusions in Table 1 in the time domain. Figure 12 shows the measured step-up waveforms of the LPF's output varied with *K_I_* and the measurement and simulation results of rising time are shown in Table 4. It can be seen that decreasing *K_I_* improves the overshoot of the control loop, but increases the rising time and decreases the settling time, which are also in accord with the simulation results.

Disturbance rejection capability (D.R.C.) is an important performance specification of the control loop, which is inversely proportional to the loop gain of the negative feedback control loop. To evaluate the D.R.C. of the control loop against system parameter variation, experiments are carried out by varying the bias voltage *V_p_* on the mass. Because the voltage *V_dc_* and the electrical-mechanical conversion parameters *K_V/C_* in Equation (6) are related to *V_p_*, changing the value of *V_p_* can imitate the system parameter variation in the control loop. As the TIA's output signal's amplitude, which reflects the resonator vibration velocity, is controlled by the negative feedback loop, the variations of *V_dc_* and *K_V/C_* will be projected on the amplitude of the drive signal. As a result, the D.R.C. of the control loop can be calculated as:

(11) $$D.R.C(dB)=20\log_{10}\left(\frac{V.P._{Dr\_\text{amp}}}{V.P._{\text{TIA}\_\text{amp}}}\right),$$

where *V.P._Dr_amp_* and *V.P._TIA_amp_* are the variation percentage of drive signal's magnitude and TIA' output signal's magnitude under different *V_p_* values.

**Figure 13.**
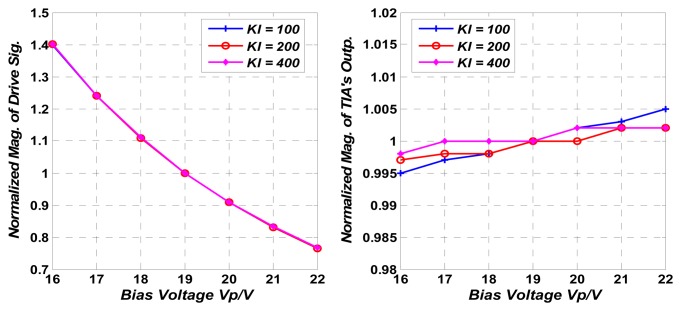
Normalized magnitude of drive signal and TIA's output with different *K_I_* by varying *V_p_*.

Both the magnitude of the drive signal and the TIA's output are recorded in Table 5 with three different values of *K_I_* and under different *V_p_* ranging from 16 V to 22 V. The percentages of variation of these two signals are calculated by setting *V_p_* = 19 V as reference and D.R.C.s are calculated from Equation (11). As shown in Table 5, the D.R.C. is increased 6 dB when *K_I_* is doubled from 100 to 200. This result shows a close agreement with both the theoretical and simulation results. When *K_I_* is increased from 200 to 400, the D.R.C. is increased only about 3.6 dB. As the minimum stable bit is the sub-mV bit in the AC magnitude measurement, it is believed that the improvement of D.R.C. is limited by the noise in the control loop. Figure 13 shows normalized magnitude of drive signal and TIA's output with different *K_I_* by varying *V_p_*. The benefits of the control loop's D.R.C. can be observed directly in Figure 13.

## 4. Conclusions

This paper presents a linearization design approach of the 3rd order velocity-controlled closed-loop for MEMS vibration gyroscope. As benefits of the built linear model, tedious stability analysis in the time domain can be transferred to the frequency domain and the design process of the control loop is simplified. The stability criterion and the mathematical expression of each performance specification of the 3rd order control loop are presented in this paper. Moreover, an optimization methodology which reveals that a robust, stable and swift control loop can be achieved by carefully selecting the system parameters following a priority order is summarized. Experiments carried out on a gyroscope prototype verify the optimization methodology that an optimized stability of the control loop can be achieved by canceling the pole of the resonator with the zero in the PI controller, and disturbance rejection capability (D.R.C) of the control loop can be improved by increasing the integral term *K_I_*. It is also found that noise in the control loop limits the D.R.C. of the control loop. As a result, the noise performance of the control loop will be studied in the future work.

## Figures and Tables

**Table 1.** Performance specifications of the control loop versus primary system parameters.

**Parameters**	**Loop Gain**	**Bandwidth**	**Phase Margin**
*K_P_*↑	↑	↑	↓
*K_I_*↑	↑	–	–
*ω_lpf_*↑	→	↑	↑
*Q*↑	↑	–	–
*K_total_*↑	↑	↑	↓

↑ Increase; ↓ Decrease; → no change; – unknown.

**Table 2.** Parameters of the control loop simulated in Matlab.

**Parameters (unit)**	**Value**	**Parameters (unit)**	**Value**
*K_F/V_^2^(N*/*V^2^)*	3.7e-8	*ω_x_(rad*/*s)*	8063 × 2π
*K_C/X_(F*/*m)*	1.2e-8	*k (N*/*m)*	29.6
*K_V/C_(V*/*F)*	2.4e12	*Q*	1,368
*K_VGA_(*/*V)*	20	*ω_lpf_(rad*/*s)*	100 × 2π
*V_ref_(V)*	0.2	*K_P_*	5
*V_p_(V)*	12	*K_I_*	200
*V_mid_(V)*	2.5	*τ*	1

**Table 3.** Comparison of simulation and measurement rise times with different proportional term *K_P_*.

	***K****_P_***= 2.5**	***K****_P_***= 5**	***K****_P_***= 10**	***K****_P_***= 20**
Simulated rise time with linear model (ms)	16.4	12.8	7.4	3.7
Simulated rise time with nonlinear model (ms)	12.0	8.6	5.1	2.8
Tested rise time (ms)	12.4	8.9	5.3	3.0

**Table 4.** Comparison of simulation and measurement rise times with different proportional term *K_I_*.

	***K****_I_***= 800**	***K****_I_***= 400**	***K****_I_***= 200**	***K****_I_***= 100**
Simulated rise time with linear model (ms)	7.3	9.7	12.8	17.0
Simulated rise time with nonlinear model (ms)	5.7	7.1	8.6	10.2
Tested rise time (ms)	5.2	7.0	8.9	10.4

**Table 5.** Measured D.R.C of the velocity-controlled closed-loop with different *K_I_* by varying *V_p_*.

**Cond.**	**V_p_(V)**	**16**	**17**	**18**	**19** *	**20**	**21**	**22**	**V.P. (%)**	**D.R.C. (dB)**
K_I_ = 100, K_P_ = 5	**Mag. of Drive Sig. (mV)**	419.8	372.0	332.8	299.8	272.6	249.6	229.8	63.4	36.2
	**Mag. of TIA's Outp. (mV)**	60.4	60.5	60.6	60.7	60.8	60.9	61.0	0.99	
K_I_ = 200, K_P_ = 5	**Mag. of Drive Sig. (mV)**	416.0	368.0	329.0	296.8	269.6	246.2	227.0	63.7	42.1
	**Mag. of TIA's Outp. (mV)**	59.8	59.9	59.9	60.0	60.0	60.1	60.1	0.5	
K_I_ = 400, K_P_ = 5	**Mag. of Drive Sig. (mV)**	416.6	369.0	330.4	297.4	270.4	247.6	227.8	63.5	45.7
	**Mag. of TIA's Outp. (mV)**	59.9	60.0	60.0	60.0	60.1	60.1	60.1	0.33	

* V.P. Variation Percentage (set *V_p_* = 19 V as reference).
